# Skeletal disproportion in glucocorticoid-treated boys with Duchenne muscular dystrophy

**DOI:** 10.1007/s00431-019-03336-5

**Published:** 2019-02-14

**Authors:** Kung-Ting Kao, Shuko Joseph, Nadia Capaldi, Sarah Brown, Marina Di Marco, Jennifer Dunne, Iain Horrocks, Sheila Shepherd, Syed Faisal Ahmed, Sze Choong Wong

**Affiliations:** 1 Developmental Endocrinology Research Group, Royal Hospital for Children, 1345 Govan Road, G51 4TF Glasgow, UK; 2 Paediatric Neurosciences Research Group, Royal Hospital for Children, Glasgow, UK; 3 Scottish Muscle Network, Queen Elizabeth University Hospital, Glasgow, UK

**Keywords:** Body proportions, Leg length, Muscular dystrophy, Sitting height, Steroid

## Abstract

We aimed to compare body segment and bone lengths in glucocorticoid-treated boys with Duchenne muscular dystrophy (DMD) with healthy controls using dual-energy absorptiometry (DXA) images. Total height (Ht), sitting height (SH), leg length (LL) and bone lengths (femur, tibia) in boys with DMD and age-matched control boys were measured using DXA**.** Thirty boys with DMD (median age 10.0 years (6.1, 16.8)) were compared with 30 controls. SH in DMD was 3.3 cm lower (95% CI − 6.1, − 0.66; *p* = 0.016). LL in DMD was 7.3 cm lower (95% CI − 11.2, − 3.4; *p* < 0.0001). SH:LL of boys with DMD was higher by 0.08 (95% CI 0.04, 0.12; *p* < 0.0001). Femur length in DMD was 2.4 cm lower (95% CI − 4.6, − 0.12; *p* = 0.04), whereas tibial length in DMD was 4.8 cm lower (95% CI − 6.7, − 2.9; *p* < 0.0001). SH:LL was not associated with duration of glucocorticoid use (SH:LL β = 0.003, 95% CI − 0.01 to 0.002, *p* = 0.72).

*Conclusion*: Glucocorticoid-treated boys with DMD exhibit skeletal disproportion with relatively shorter leg length and more marked reduction of distal long bones. As glucocorticoid excess is not associated with such disproportion, our findings raise the possibility of an intrinsic disorder of growth in DMD.

**Table:** 

**What is Known** *• Severe growth impairment and short stature are commonly observed in boys with Duchenne muscular dystrophy (DMD), especially those treated with long-term glucocorticoids (GC).* *• In other groups of children with chronic conditions and/or disorders of puberty, skeletal disproportion with lower spinal length has been reported.*	
**What is New** • *Growth impairment in GC-treated boys with DMD was associated with skeletal disproportion in relation to age, with lower limbs and distal long bones affected to a greater degree.*

**Electronic supplementary material:**

The online version of this article (10.1007/s00431-019-03336-5) contains supplementary material, which is available to authorized users.

## Introduction

Severe growth impairment and short stature are commonly observed in boys with Duchenne muscular dystrophy (DMD), especially those treated with long-term glucocorticoids (GC). Several observations point to the possibility that DMD, itself, may be associated with a growth disorder. Approximately, a quarter of boys with DMD are short prior to initiation of GC [1–3], and growth failure that pre-dates the initiation of GC therapy has been reported [4]. It is increasingly recognised that the majority of adolescent boys with DMD who continue on GC may have persistent hypogonadism. In other groups of children with chronic conditions and/or disorders of puberty, skeletal disproportion with lower spinal length has been reported [5, 6]. Mechanical stimulation may play a role in stimulating growth, as inferred from studies in children with hemiplegic cerebral palsy where leg length discrepancy has been reported [7]. Whilst experimental studies using in vitro and in vivo models allow evaluation of longitudinal bone growth [8, 9], clinical studies of bone length and body proportions in DMD are not available to the best of our knowledge.

Annual monitoring of bone density using dual-energy X-ray absorptiometry (DXA) is part of standard care in DMD [10]. Total body DXA scans can be analysed using digital analysis tools in the DXA machine to allow measurements of body proportions and bone lengths, similar to the measurement of vertebral height for vertebral morphometry [11]. The use of DXA to assess body proportion and measure bone length has been shown to be accurate and precise in adults [12], and recently confirmed by our group to be feasible in children with chronic conditions [13].

The primary aim of this study is to compare body segments and bone lengths in boys with DMD treated with GC in comparison with a group of age-matched healthy controls using DXA total body images.

## Materials and methods

Of the 41 boys with DMD recruited into a prospective longitudinal study of bone morbidity between January 2016 and March 2017, 30 who were not GC naïve, who had not discontinued GC or did not have any metal instrumentation and/or severe scoliosis based on a Cobb angle of greater than 20 degrees and had DXA performed on the Lunar iDXA (GE Lunar Corp, Madison, WI, USA) were included in this present study. Contractures were defined by the presence of hip extension greater than − 10 degrees and/or knee extension greater than − 10 degrees from neuromuscular physiotherapist assessment as per NorthStar Assessment Guidelines. NorthStar Ambulatory Assessment (NSAA) scores were reported for those who were ambulant, based on structured physiotherapy assessment at study visits [14]. NSAA scores range from 0 to a maximum score of 34. Pubertal assessment was performed by a single independent assessor (SCW) using the Tanner and Whitehouse method [15]. Vertebral fractures were diagnosed from DXA lateral vertebral morphometry.

Thirty out of 94 healthy locally recruited age-matched boys who had DXA performed as part of a normative study of DXA bone mineral density was the comparative group [16]. Controls were individually age matched to be within 6 months in age with the DMD group. DXA in the healthy cohort was performed on the Lunar Prodigy (GE Medical Systems, Waukesha, Wisconsin, USA). Pubertal assessment in this group was performed using self-assessment forms.

Both studies were approved by the West of Scotland ethics committee. Each parent and participant provided written informed consent/assent prior to study enrolment.

### Body segment and bone length measurements using DXA images of the total body measurement

Body segments (sitting height, total height, lower limb length, vertebral column length and upper limb length), body proportion (sitting height to lower limb ratio) and bone lengths (femur, tibia, humerus and forearm) were measured using DXA images. Measurements were performed in triplicate on different occasions, by a single observer (KTK), blinded to results from previous measurements. The methodology for measuring sitting height, total height and lower limbs using DXA has been previously reported (Supplementary Fig. 1a) [13]. The measurement with the greatest amount of difference from the median value of the three measurements for each subject was excluded. The remainder two measurements were averaged and used for analysis.

For images where the subject had abducted legs and equinus foot position (“Bent position”), leg length was defined by measurements of bone lengths. The distances between the ischium and condyles, condyles and the talus, and the talus to the sole of the foot were measured for each lower limb. The average of measurements of both lower limbs was taken as the leg length (Supplementary Fig. 1b).

Vertebral column was defined as the distance from the sternum (T2) to the top of the iliac crest (L4). Femur length was defined as the distance from the femoral head to the condyles. Tibia length was defined as the measurement between the condyles and the mortise joint. Humerus length was defined as the measurement from the top of the humeral head to the base of epicondyles. Forearm bone length was defined as the measurement from the radial head and radial/ulna styloid processes. The sum of the humerus and forearm was taken as the upper limb length (Supplementary Fig. 1c).

### Statistical analyses

All statistical analysis was performed using IBM SPSS 21 (SPSS Inc., Chicago). Results were expressed as median (range) given the relatively small sample size, and the majority of the data were not normally distributed.

Two-way mixed effect intraclass correlation (ICC) was used to evaluate the intra-observer agreement between body proportion and bone length measurements. Relative technical error of measurement (rTEM) was calculated using the remaining two DXA measurements, following removal of the measurement with the greatest amount of difference from the median value of the three measurements. Differences between body proportions and bone lengths in DMD and healthy controls were evaluated using linear regression following adjustment pubertal status (pre-pubertal vs pubertal—pre-pubertal, i.e. Tanner stage 1 as reference category). *p* < 0.05 was considered statistically significant.

## Results

### Demographics

Table 1 shows demographics of the boys with DMD and healthy controls. There were no differences in age between the two groups. Almost 60% of the healthy controls were pre-pubertal, whereas the 26 (87%) boys with DMD were pre-pubertal. Two (6.7%) boys with DMD were in Tanner stages 2/3, and two (6.7%) were in Tanner stages 4/5. Boys with DMD were shorter and had a higher body mass index (BMI) SDS compared with healthy controls (Table 1). Two of the boys with DMD were on testosterone therapy for a duration of 1.5 and 2.0 years at the study visit. None were on growth hormone therapy. Out of the 30 boys with DMD, 20 had no hip and knee contractures, all of whom were still ambulant.

**Table 1:** Baseline demographics of boys with DMD and healthy controls

	Controls	DMD	*p* value
	(*n*, 30)	(*n*, 30)	
Age	10.2 (6.3–16.9)	10.0 (6.1–16.8)	0.97
Pubertal stage (%)			0.02
Pre-pubertal	17 (57%)	26 (87%)	
Pubertal	13 (43%)	4 (13%)	
Height SDS	+ 0.3 (− 1.4 to + 2.5)	− 1.7 (− 7.0 to + 1.7)	< 0.001
BMI SDS	+ 0.6 (− 1.3 to + 1.8)	+ 2.0 (− 1.4 to + 4.0)	< 0.001
Steroid duration (years)		7.1 (1.3 to 15.2)	–
Steroid dose* (mg/m2/day)		72.7 (21.1–184.3)	–
Pulsed steroid treatment		3 (10%)	–
Testosterone treatment		2 (7%)	–
Bisphosphonate		10 (33%)	–
Vertebral fractures		14 (47%)	–
Non-ambulant		10 (33%)	–
NSAA score **		26.5 (8 to 33)	
Hip/knee contractures		10 (33%)	
Bone age (years)		9.5 (5.0–16)	–

*DMD*, Duchenne muscular dystrophy; *SDS*, standard deviation score; *BMI*, body mass index; *NSAA*, NorthStar Ambulatory Assessment

Results are presented as median (range)

*Steroid dose reported as hydrocortisone equivalent: prednisone 1 mg = hydrocortisone 4 mg; deflazacort 1 mg = 6 mg hydrocortisone. Dose is half if the patient is on pulsed treatment

**Maximal score of NSAA score is 34 and consists of 17 domains of lower limb muscle function. The assessments are performed by trained neuromuscular physiotherapist and are results of the 20 ambulant boys with DMD

### Reproducibility of DXA measurements

A high degree of reproducibility was found for all measurements in both the DMD and control groups, with ICC ranging between 0.927 and 0.998 in controls and 0.975 and 0.999 in boys with DMD (Supplementary Table 1). Intra-observer variability of the remaining two measurements showed excellent agreement with the rTEM below 1% for all measurements. rTEM for tibia and forearm measurements in the DMD group was 1.2%, respectively.

### Body segments and body proportion in DMD compared with controls

**Fig. 1:**
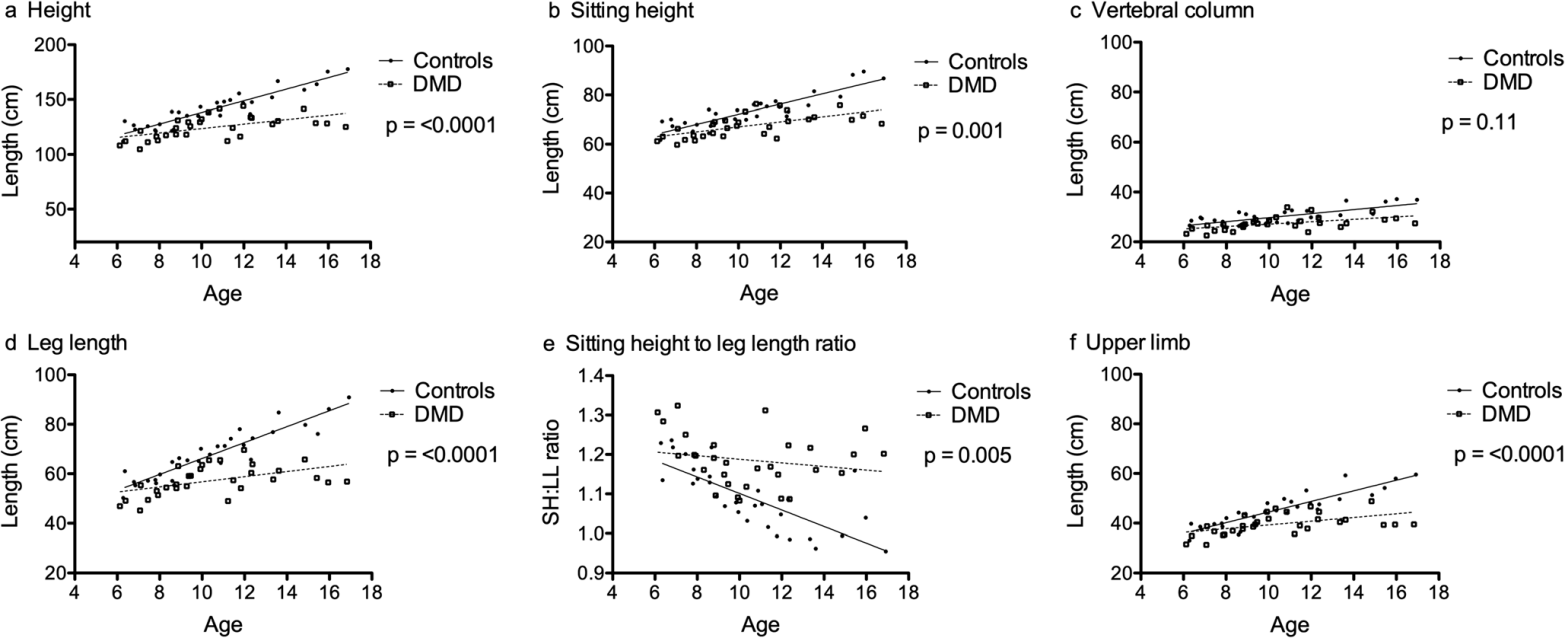
Anthropometry of boys with DMD in comparison with healthy controls (unadjusted analysis). **a** Ht in boys with DMD compared with controls. **b** SH in boys with DMD compared with controls. **c** VC in boys with DMD compared with controls. **d** LL in boys with DMD compared with controls. **e** SH:LL ratio in boys with DMD compared with controls. **f** UL in boys with DMD compared with controls. Statistical analysis was performed using linear regression analysis. Empty squares represent boys with DMD and solid circles represent healthy control boys. Solid lines represent the lines of best fit. *p* values are for differences between DMD and control *β* slopes. DMD, Duchenne muscular dystrophy; Ht, total height; SH, sitting height; VC, vertebral column; LL, leg length; UL, upper limb

Height and body segments were lower in DMD compared with healthy controls in unadjusted analysis (Fig. 1a–f). Body proportion as assessed by sitting height to leg length ratio was higher in DMD compared with healthy controls in unadjusted analysis (Fig. 1e).

Table 2 shows the results of height and body segments in boys with DMD compared with healthy controls after adjusting puberty. The height of boys with DMD was 10.7 cm lower (95% CI − 17.1 to − 4.3). Sitting height and vertebral column were 3.3 cm (95% CI − 6.1 to − 0.66) and 1.7 cm lower (95% CI − 3.2 to − 0.31), respectively. In contrast, the leg length of boys with DMD was 9.9 cm lower (95% CI − 13.1 to − 6.6). Median percentage difference for sitting in boys with DMD in comparison with controls was − 6.5% (− 24% to + 6.7%). In contrast, median percentage difference for leg length in boys with DMD in comparison with controls was − 13% (− 46% to + 13%). Sitting height to leg length ratio of boys with DMD was higher by 0.08 (95% CI 0.04 to 0.12). The upper limb of boys with DMD was 3.6 cm lower (95% CI − 6.2 to − 0.97).

**Table 2:** Body proportion and bone length differences between boys with DMD and controls (adjusted model)

	*β* (95% CI)	*r* ^2^	*p* value
Body proportions			
SH	− 3.3 (− 6.1 to − 0.66)	0.44	0.016
LL	− 7.3 (− 11.2 to − 3.4)	0.48	< 0.0001
UL	− 3.6 (− 6.2 to − 0.97)	0.45	0.008
VC	− 1.7 (− 3.2 to − 0.31)	0.38	0.018
Height	− 10.7 (− 17.1 to − 4.3)	0.49	0.001
SH:LL	0.08 (0.04 to 0.12)	0.36	< 0.0001
Bone lengths			
Femur	− 2.4 (− 4.6 to − 0.12)	0.37	0.04
Tibia	− 4.8 (− 6.7 to − 2.9)	0.56	< 0.0001
Humerus	− 0.81 (− 2.3 to 0.64)	0.33	0.268
Forearm	− 2.7 (− 4.0 to − 1.3)	0.54	< 0.0001

*DMD*, Duchenne muscular dystrophy; *SH*, sitting height; *LL*, leg length; *UL*, upper limb; *VC*, vertebral column

Linear regression models were constructed with pubertal status (pre-pubertal vs pubertal—pre-pubertal as reference category) and disease category (control vs DMD—control as reference category) as independent factors

Sub-analysis of the 20 boys with DMD with no hip and/or knee contractures with 20 age-matched healthy controls showed identical results in similar adjusted regression models. The height of the subset of these boys with DMD was 11.5 cm lower (95% − 1.97 to − 3.4, *p* = 0.007). Sitting height and vertebral column of these boys with DMD were 4.2 cm (95% CI − 7.7 to − 0.69, *p* = 0.02) and 2.4 cm lower (95% CI − 4.2 to − 0.56, *p* = 0.012), respectively. Similarly, leg length of these boys with DMD was 7.4 cm lower (95% CI − 12.4 to − 2.4, *p* = 0.005). Median percentage difference for sitting height in this subset of boys with DMD was − 9.2% (− 24% to + 6.8%). In contrast, median percentage difference for leg length in this subset of boys with DMD was − 16% (− 46% to + 1.7%) and the sitting height to leg length ratio of these boys with DMD was higher by 0.07 (95% CI 0.02 to 0.12).

### Bone lengths in DMD compared with controls

**Fig. 2:**
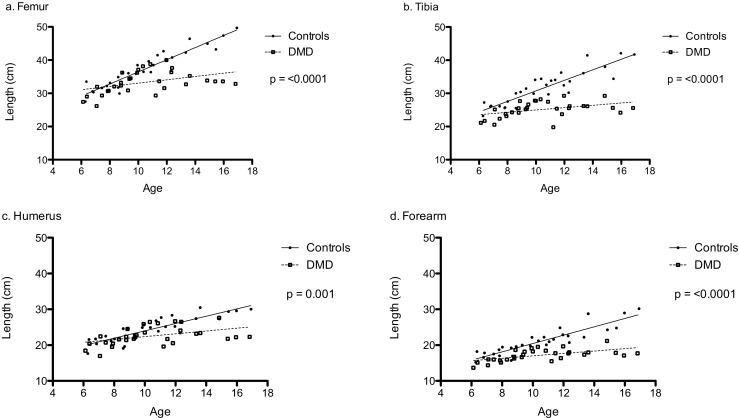
Bone lengths of boys with DMD in comparison with healthy controls (unadjusted analysis). **a** Femur length in boys with DMD compared with controls. **b** Tibial length in boys with DMD compared with controls. **c** Humerus length in boys with DMD compared with controls. **d** Forearm length in boys with DMD compared with controls. Statistical analysis was performed using linear regression analysis. Empty squares represent boys with DMD and solid circles represent healthy control boys. Solid lines represent the lines of best fit. *p* values are for differences between DMD and control *β* slopes. DMD, Duchenne muscular dystrophy

All bone lengths were lower in DMD compared with healthy controls in unadjusted analysis (Fig. 2).

Table 2 shows the results of bone lengths in boys with DMD compared with healthy controls after adjusting for puberty. Femur and tibia lengths of boys with DMD were 2.4 cm (95% CI − 4.6 to − 0.12] and 4.8 cm lower (95% CI − 6.7 to − 2.9) respectively. Median percentage difference for femur and tibial length in boys with DMD in comparison with controls were − 12% (− 41% to + 19%) and − 23% (− 53% to + 9.4%), respectively. Forearm bone lengths of boys with DMD were 2.7 cm lower (95% CI − 4.0 to − 1.3). No significant difference was observed in humerus length in boys with DMD compared with controls. Median percentage difference for humerus and forearm bone lengths in boys with DMD in comparison with controls was − 7.5% (− 34% to + 22%) and − 16% (− 52% to + 9.6%), respectively.

Similarly, sub-analysis of the 20 boys with DMD with no hip and/or knee contractures with 20 age-matched healthy controls showed identical results for bone lengths using adjusted regression models. Tibia lengths of this subset of boys with DMD were 4.4 cm lower (95% CI − 6.8 to − 2.0, *p* = 0.001). No significant difference was observed in femur length in boys with DMD compared with controls. Median percentage difference for tibial and femur lengths in this subset of boys with DMD in comparison with controls was − 21% (− 54% to + 1.3%) and − 8.4% (− 41% to + 7.6%), respectively. Median percentage difference for humerus and forearm bone lengths in this subset of boys with DMD in comparison with controls was − 7.2% (− 32% to + 13%) and − 16% (− 52% to − 1.4%), respectively.

### Association of sitting height to leg length ratio in DMD with GC and mobility score

No association was observed between duration of GC and sitting height to leg length ratio (*β* = 0.003, 95% CI − 0.01 to 0.002, *p* = 0.72) and dose of GC in hydrocortisone equivalent dose (*β* = 0.00, 95% CI − 0.0001 to 0.001, *p* = 0.09), adjusted for age and pubertal status. No association was observed between NSAA score at study visit and body disproportion (*β* = − 0.002, 95% CI − 0.006 to − 0.002, *p* = 0.23) in the subset of boys without contractures, adjusted for age and pubertal status.

## Discussion

This study used DXA total body images to evaluate body segments and bone lengths and demonstrated for the first time that skeletal disproportion exists in boys with DMD treated with GC. In these boys, the deficit was greater in the lower limbs compared with the spine. Furthermore, distal long bones in the lower and upper limb were more affected in DMD compared with healthy controls.

In our present report, we showed that measurement of bone lengths in DMD is feasible using total body images from DXA scans, extending our recent report in children with chronic conditions [13]. The Lunar GE iDXA machine, used in our current study, utilises a narrow-angle fan X-ray beam to obtain images. Fan beams, such as the wide fan beam used in Hologic machines may lead to distortion of images in up to 37% of bone mineral density measurements, which may lead to parallax errors [17, 18]. However, Lunar DXA machines use a multi-view image reconstruction technique to reduce distortion and reduce magnification errors [19]. Parallax errors in the DXA images are an issue with measurement of height using this method as some boys with DMD may have hip and knee contractures and are therefore unable to lie flat. In our sub-analysis of those who did not have lower limb contractures, greater impairment in the lower limb and distal bones was also observed, demonstrating that our observations in the whole group were not due to image distortion caused by lower limb contractures.

Short stature is a prominent feature in boys with DMD, with normal length observed at birth, followed by a gradual deceleration in growth after the first few years of life prior to the introduction of GC [2]. Although there is no doubt that short stature is exacerbated by the use of GC in DMD [20, 21], supraphysiological doses of GC in juvenile arthritis do not lead to skeletal disproportion [22]. In our study, no relationship was observed between glucocorticoid duration and dose with body proportion after adjusting for age and pubertal status. This provides further evidence that factors other than GC may play a role in the disproportion observed in our study. Future studies should evaluate body segments in DMD prior to the initiation of GC and following therapy. Clinical practice over the last decade and in accordance with the international standards of care recommends the introduction of GC in DMD from approximately 3–4 years, an age where DXA for bone monitoring is often not performed due to lack of normative bone mineral density data in those < 5 years [23]. In our clinic of 51 boys with DMD in 2018, none is GC naive.

The underlying genotype may be an explanation for the skeletal disproportion in DMD. Children with short stature homeobox-containing gene (SHOX) deletion are known to have disproportionately shorter leg length and distal long bone abnormalities [24]. SHOX is exclusively expressed in the first and second pharyngeal arches and in the developing distal limb bones of human embryos [25], resulting in compromised linear growth and unbalanced premature growth plate fusion of the distal limb bones [26, 27]. The dystrophin gene is located in the Xp21 region, adjacent to the *SHOX* gene, which is located in the pseudoautosomal region of Xp22. It is possible that the molecular defects in the Xp21 region may also involve adjacent genes in the Xp22 region, causing the abnormal lower limb growth patterns [2]. However, the deletion of Xp22 as part of a contiguous gene deletion syndrome is only found in a minority of boys with DMD [28]. Two studies in DMD have identified that short stature is more common in boys with DMD with distal deletions suggesting a genotype effect on linear growth in DMD [1, 29]. Both studies did not evaluate body segments.

Another possible explanation of the observation of disproportionately shorter legs in DMD is the lack of mechanical stimulation due to the underlying myopathy. Postnatal bone growth is dependent on an intact ‘muscle-bone’ unit, and the development of children’s load-bearing bones depends strongly on muscle mass and strength [30]. In healthy children, earlier age of walking predicts greater bone mass in later childhood and adolescence, although these studies did not evaluate the impact on longitudinal bone growth [31, 32]. In rat models, osteocytes at the endosteal side of the shaft and the inner lamellae are mechanosensitive. The absence of mechanical load in these animal models resulted in decreased bone formation and longitudinal bone growth via a reduction in local IGF-1 production [33–35]. Studies in children with cerebral palsy showed that tibial length is reduced by up to 5 cm, with greater reduction in tibial length in those with more severe CP and increasing age [36]. In our sub-analysis, we did not find an association with the NSAA scores and disproportion at the study visit. However, longitudinal assessments of muscle function are required to provide greater insight into the relationship with growth in the lower limb of boys with DMD, as reduction in weight-bearing activity in these boys may contribute in part to our observations.

There are several limitations of our current study with a relatively small sample size. The cross-sectional nature of the study precluded us from meaningful analysis and interpretation of disease and treatment factors on bone length and disproportion. Images of the DMD and control groups were performed using different Lunar DXA machines. However, repeatability of measurements in both DMD and healthy controls was in the excellent category. As BMD correlation between these two instruments was shown to be excellent with comparable precision previously, we believe that this should not introduce significant intergroup bias [37]. Pubertal assessment was not performed in the similar manner in the boys with DMD and healthy controls. However, we believe our study identified new insights into growth impairment in DMD and future studies to elucidate the underlying mechanisms are now needed.

## Conclusion

This study showed for the first time that growth impairment in GC-treated boys with DMD was associated with skeletal disproportion, with lower limbs being affected to a greater degree compared with the spine, and that distal long bones were more affected. Further, the disproportion appears to increase with age. As skeletal disproportion is not a known finding with paediatric GC excess, our observation of disproportionate bone lengths in boys with DMD raises the question of whether DMD is an intrinsic and localised disorder of growth or diminished growth of the extremities secondary to the myopathy, which may be best investigated in future experimental studies.

## Electronic supplementary material

ESM 1 (DOCX 2735 kb)

ESM 2 (DOCX 15 kb)

## Abbreviations

DMD: Duchenne muscular dystrophy
DXA: Dual-energy X-ray absorptiometry
GC: Glucocorticoids
Ht: Total height
LL: Leg length
NSAA: NorthStar Ambulatory Assessment
SDS: Standard deviation score
SH: Sitting height
SHOX: Short stature homeobox-containing gene
VC: Vertebral column
