# Supplementary material for: Skeletal disproportion in glucocorticoid-treated boys with Duchenne muscular dystrophy
Source: Eur J Pediatr. 2019 Feb 14;178(5):633–40. doi: 10.1007/s00431-019-03336-5 (PMC6459782; doi:10.1007/s00431-019-03336-5)
Supplement: Supplementary file 1 — (DOCX 2735 kb) [file 431_2019_3336_MOESM1_ESM.docx]

Supplementary Figure 1a

**Protocol of DXA measurement of height, sitting height, leg length and tibia.**

Sitting Height measurements

Draw a perpendicular line (line 6) between the horizontal line at the top of the skull (line 1) to the horizontal line at the bottom of ischium (line 2).

Lower Limb measurements

Draw a perpendicular line (line 7) between the horizontal line at the bottom of ischium (line 2) to the horizontal line at the bottom of the calcaneus (line 5).

Height measurements

Height = Sitting Height + Lower Limb

Tibia measurements

Tibia - Perpendicular line (line 8) between the horizontal line at the knee joint (line 3) to the horizontal line at the mortise joint (line 4).

**
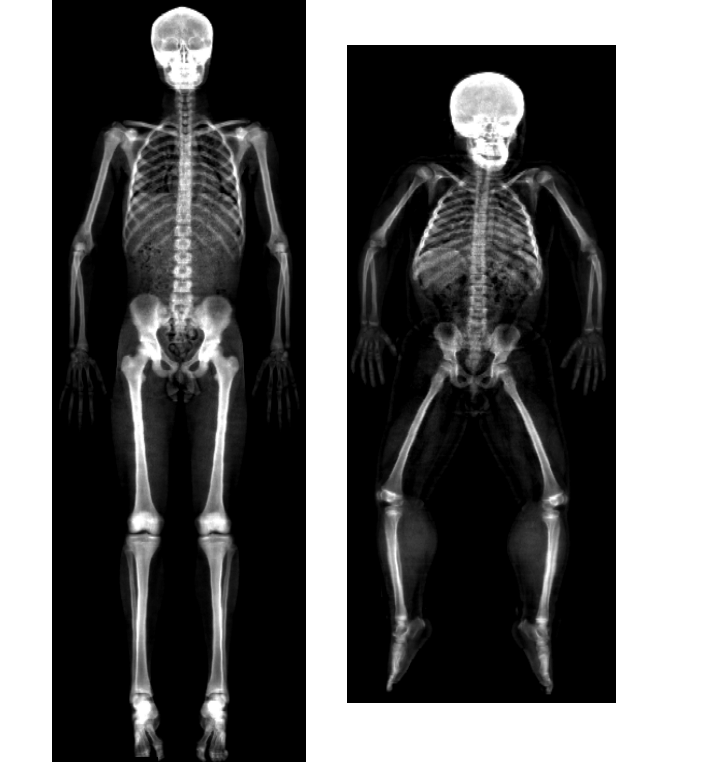
**

**1**

**7**

**8**

**2**

**3**

**4**

**5**

**6**

**Supplementary figure 1a**

Supplementary Figure1b

**Protocol of DXA measurement of leg length and tibia with abducted legs and equinus foot position**

Sitting height measurement as per Supplementary Figure 1a.

Lower limb measurements

(b): Measure line parallel to the femur (line 9) from the bottom of ischium (line 2) to the line at the femoral and tibial condyles (line 6).

(c): Measure line parallel to the tibia (line 10) joining line 6 to the top of talus (line 3).

(d): Measure perpendicular line (line 11) from intersect of line 3 and line 7 to line 8 (parallel to sole of foot).

Measure right and left lower limb as above.

LL = (b) + (c) + (d)

Take the average of the right and left leg measurement as leg length measurement.

Ht = SH + LL

Tibia measurements

Tibia - Perpendicular line (line 10) between the horizontal line at the knee joint (line 6) to the horizontal line at the top of the talus (line 3).


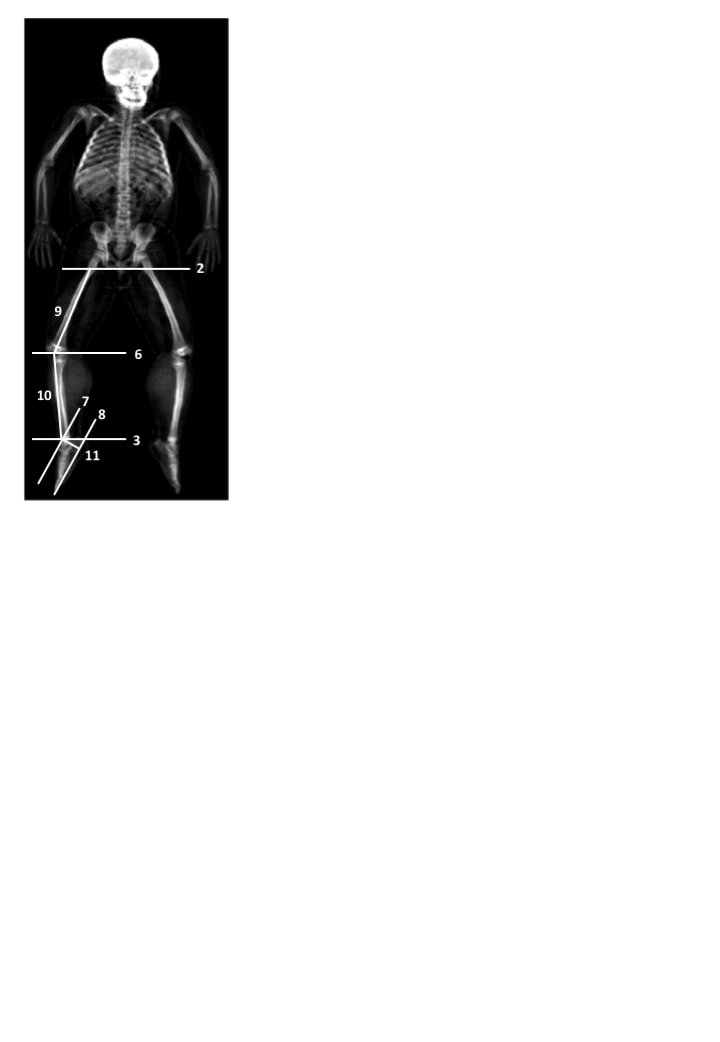


**Supplementary figure 1b**

Supplementary Figure 1c

**Protocol for Vertebral column, femur, humerus and forearm measurements.**

Vertebral column measurements

Find the sternum (Thoracic spine T1-2) by drawing lines (Lines 1, 2) along the medial ends of both clavicles until both lines cross

Draw a horizontal line (Line 3) across the top of the iliac crests (Lumbar spine L4).

Measure the vertebral column (line 4) by drawing a perpendicular line from the sternum (Line 1 and 2 intersection) to the iliac crest line (Line 3).

Femur measurements

Draw a horizontal line across the top of the femoral head in the acetabulum (Line 5)

Draw a horizontal line between femoral and tibial condyles (Line 6)

Measure the femur length (Line 7) by drawing a perpendicular line between Line 4 and 5.

*Upper Limb, Humerus and Forearm measurements*

Humerus measurement

Draw a horizontal line across the top of the humerus in the shoulder (Line 8)

Draw horizontal line across elbow joint. (Line 9)

Measure the humerus by drawing a line between Line 8 and 9 along the humerus (Line 11).

Forearm measurement

Draw a horizontal line across the top of the wrist joint (Line 10)

Measure the forearm by drawing a perpendicular line between Line 9 and 10. (Line 12)

Upper Limb = Humerus + Forearm


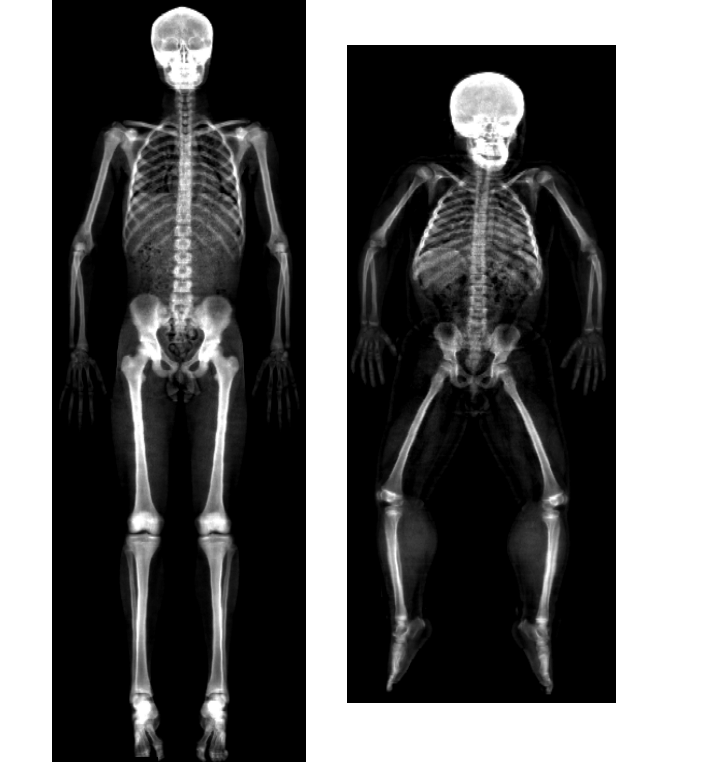


1

2

3

4

6

5

7

10

9

8

12

11

**Supplementary figure 1c**
