# Supplementary material for: Skeletal disproportion in glucocorticoid-treated boys with Duchenne muscular dystrophy
Source: Eur J Pediatr. 2019 Feb 14;178(5):633–40. doi: 10.1007/s00431-019-03336-5 (PMC6459782; doi:10.1007/s00431-019-03336-5)
Supplement: Supplementary file 2 — (DOCX 15 kb) [file 431_2019_3336_MOESM2_ESM.docx]

**Supplementary Table 1: Intraclass correlation coefficient and relative technical error of measurements for body proportion and bone length measurements in boys with DMD and controls.**

|  | **Controls** | | | **DMD** | | | |
| --- | --- | --- | --- | --- | --- | --- | --- |
|  | **ICC** | **95% CI** | **rTEM (%)** | **ICC** | **95% CI** | **rTEM** |  |
| **Body proportions** |  |  |  |  |  |  |  |
| **Sitting Height** | 0.927 | 0.907 to 0.944 | 0.16% | 0.996 | 0.992 to 0.998 | 0.25% |  |
| **Leg length** | 0.952 | 0.939 to 0.963 | 0.25% | 0.999 | 0.999 to 1.000 | 0.59% |  |
| **Vertebral column** | 0.976 | 0.966 to 0.984 | 0.79% | 0.982 | 0.967 to 0.990 | 0.87% |  |
| **Bone lengths** |  |  |  |  |  |  |  |
| **Femur** | 0.998 | 0.997 to 0.998 | 0.42% | 0.975 | 0.956 to 0.987 | 1.50% |  |
| **Tibia** | 0.994 | 0.992 to 0.995 | 0.61% | 0.981 | 0.966 to 0.990 | 1.21% |  |
| **Humerus** | 0.997 | 0.996 to 0.998 | 0.99% | 0.993 | 0.987 to 0.996 | 0.76% |  |
| **Forearm** | 0.991 | 0.988 to 0.994 | 0.99% | 0.984 | 0.972 to 0.992 | 1.2% |  |

DMD: Duchenne muscular dystrophy; ICC: intraclass correlation coefficient; rTEM: relative technical error of measurement.

Note: ICC and rTEM for height and upper limb not calculated, as they are composites of sitting height and leg length or humerus and forearm lengths, respectively.
